# Recombinant IFN-α2a-NGR exhibits higher inhibitory function on tumor neovessels formation compared with IFN-α2a in vivo and in vitro

**DOI:** 10.1007/s10616-014-9743-y

**Published:** 2014-06-05

**Authors:** Weina Li, Qiang Hao, Liqing He, Jieru Meng, Meng Li, Xiaochang Xue, Cun Zhang, Hong Li, Wei Zhang, Yingqi Zhang

**Affiliations:** 1 The State Key Laboratory of Cancer Biology, Department of Biopharmaceutics, School of Pharmacy, Fourth Military Medical University, Xi’an, 710032 People’s Republic of China; 2 Laboratories of Respiratory Biology, National Institute of Environmental Health Sciences, National Institutes of Health, Research Triangle Park, NC 27709 USA; 3 Department of Biopharmaceutics, School of Pharmacy, The Fourth Military Medical University, 17 Changle West Road, Xi’an, 710032 People’s Republic of China

**Keywords:** IFN-α2a-NGR, Tumor-targeted IFN-a2a, Anti-tumor activity, NGR peptide, Tumor neovessels

## Abstract

We previously reported that NGR-fused IFN-α2a (IFN-α2a-NGR) exhibited similar biological activities with native IFN-α2a and was well-tolerated in mice, rats and monkeys. In the current study, we evaluated the mechanisms of this fusion protein on angiogenesis and tumor formation. Our data indicated that IFN-α2a-NGR has the ability to target tumor blood vessels while preserving the original function of native IFN-α2a. IFN-α2a-NGR was found to be concentrated in the tumor tissues, particularly around the vessel areas. In contrast to IFN-α2a, IFN-α2a-NGR significantly decreased microvessel density and increased the apoptosis of vascular endothelial cells. IFN-α2a-NGR also decreased the expression of VEGF and bFGF in tumor cells. Significant inhibition of invasion, migration, tube formation and induction of apoptosis of endothelial cells were observed in IFN-α2a-NGR-treated group. In conclusion, results from in vitro and in vivo experiments indicate that IFN-α2a-NGR is a promising anti-angiogenic agent with greater therapeutic efficacy than IFN-α2a.

**Electronic supplementary material:**

The online version of this article (doi:10.1007/s10616-014-9743-y) contains supplementary material, which is available to authorized users.

## Introduction

Interferon-alpha2a (IFN-α2a) is a multifunctional cytokine that has anti-viral, immunomodulatory and anti-tumor effects (Petska et al. 1987). The anti-tumor effects of IFN-α2a are mediated through upregulation of surface expression of major histocompatibility complex (MHC) class I molecules, enhancement of the proliferation of type-I helper T cells (Th1) (Belardelli 1995) and generation of cytotoxic T lymphocytes (CTLs) in specific antitumor immune responses (von Hoegen et al. 1990). IFN-α2a has also been shown to inhibit of cell growth and antiangiogenesis (Okada et al. 2001) and directly inhibit the release of tumor-derived pro-angiogenic factors such as VEGF and bFGF (von Marschall et al. 2003; Singh et al. 1995).

Because of its various therapeutic properties, IFN-α therapy has been approved worldwide for the treatment of various malignancies and virological diseases, such as hairy cell leukemia, melanoma, and renal cell carcinoma (Rios et al. 1985). However, IFN-α2a therapy is associated with significant toxicities, including severe neuropsychiatric, hematologic and hepatic effects (Jonnasch and Haluska 2001).

Several tumor-homing peptides, such as the cyclic peptide, NGR (CNGRCVSGCAGRC), have been found in vivo and were used in combination with anti-cancer drugs to increase the anti-tumor activity of these drugs and reduce the toxicity to normal tissues (Arap et al. 1998; Sacchi et al. 2004; Wang et al. 2006).

In previous studies, we constructed the fusion protein IFN-α2a-NGR. We coupled an NGR-containing peptide (GNCNGRCVSGCAGRC) with the C-terminal of IFN-a2a and expressed the fusion protein in *E. coli.* Purification was achieved via ion exchange chromatography (Meng et al. 2007). Preclinical safety studies revealed that IFN-α2a-NGR was well tolerated at pharmacologically active doses in mice, rats and monkeys (Meng et al. 2008).

Here, we further investigate the effects of this fusion protein on tumor growth and anti-angiogenic activity. Our data indicated that IFN-α2a-NGR has a greater inhibitory effect on the growth of tumors compared to that of IFN-α2a. IFN-α2a-NGR increased the anti-tumor activity of IFN-α2a and lowered the dosage required for this effect. Our findings indicated that IFN-α2a-NGR mediated its anti-tumor effects by decreasing pro-angiogenic factors and inhibiting the function of vascular endothelial cells.

## Materials and methods

### Animals

Healthy female nude mice (4–6 weeks old) were purchased from the Institute of Pharmaceutical Research Animal Resource of Shanghai and maintained under institutional animal care protocols.

### Cell culture

A549 (human lung cancer cell line) and SPC-A-1 (human lung cancer cell line) cells were cultured in RPMI 1640 (Gibco, Grand Island, NY, USA). Human umbilical vein endothelial cells (HUVEC) were cultured in DMEM (Gibco) supplemented with 10 % fetal bovine serum (Hyclone, Logan, UT, USA), 2 mM glutamine, 100 U/mL penicillin and 100 mg/L streptomycin. All cells were maintained at 37 °C in a 5 % CO_2_ incubator. All cell lines were purchased from the National Rodent Laboratory Animal Resource (Shanghai, China).

### Expression and purification of IFN-a2a-NGR

The IFN-α2a-NGR was purified and prepared as previously reported (Meng et al. 2007). In brief, IFNα2a-NGR was produced by a strain of *Escherichia coli* bearing a genetically engineered plasmid that contains the IFNα2a-NGR gene and was purified to homogeneity by a combination of Q-Sepharose and SP-Sepharose fast-flow chromatography.

### Tumor implantation and anti-tumor studies in vivo

A549 or SPC-A-1 cells (1 × 10^7^ cells/mouse) were injected subcutaneously into healthy nude mice. 10 days after transplantation, as the mean size of the tumors was about 0.4 cm in diameter, mice with size-matched tumors were randomized into seven treatment groups (n = 8): 0.9 % sodium chloride group, low dose (LD) IFN-α2a-NGR group (1 × 10^6^ IU/kg), middle dose (MD) IFN-α2a-NGR group (3 × 10^6^ IU/kg), high dose (HD) IFN-α2a-NGR group (9 × 10^6^ IU/kg), LD IFN-α2a group (1 × 10^6^ IU/kg), MD IFN-α2a group (3 × 10^6^ IU/kg) and HD IFN-α2a group (9 × 10^6^ IU/kg). The purified IFN-α2a was purchased from Santa Cruz Biotechnology (Santa Cruz, CA, USA; sc-4623). All drugs, diluted with 0.9 % sodium chloride, were administered i.p. once a day. Animals were sacrificed when the tumors reached 1.8–2.0 cm in diameter and the inhibition rates of the growth of A549 or SPC-A-1 xenografted tumors were calculated according to the formula: inhibition rate (%) = (1 − tumor weight in test group/tumor weight in control group) × 100.

### ELISA assay

The microtiter plates were coated with purified recombinant human CD13 at 1 μg/well (Sino Biological Inc, Beijing, China) at 4 °C overnight. Plates were then washed with PBS containing 0.1 % Tween 20 and the remaining protein-binding sites were blocked with PBS containing 0.1 % Tween 20 and 5 % non fat dry milk prior to the addition of serially diluted IFN-α2a-NGR or IFN-α2a at RT for 2 h. Plates were then washed and the bound IFN-α2a-NGR or IFN-α2a was detected using an anti-hIFN-α monoclonal antibody (Abcam, Cambridge, UK). The reaction was developed with o-phenylenediamine as the substrate and measured with a Bio-Rad ELISA reader (OD490) (Hercules, CA, USA).

### Total RNA extraction and Real-time PCR

Total RNA from tumors were isolated using Trizol reagent (Invitrogen Life Technologies, Carlsbad, CA, USA) according to the manufacturer’s instructions. First-strand complementary DNA synthesis was performed using the cDNA RT Kit (Takara, Otsu, Shiga, Japan). Portions of double-stranded cDNA were subjected to PCR with a SYBR Green PCR Reagents kit (Applied Biosystems, Foster City, CA, USA). PCR was performed at 95 °C for 15 min, followed by 35 cycles of (95 °C for 20 s, 56 °C for 30 s, 72 °C for 30 s), followed by 72 °C for 1 min. The following primer pairs were used: VEGF forward 5′-AAGCCATCCTGTGTGCCCCTGATG-3′, reverse 5′-GCGAATTCCTCCTGCCCGGCTCAC-3′; bFGF forward 5′-CTGTACTGCAAAAACGGG-3′, reverse 5′-AAAGTATAGCTTTCTGCC-3′; and β-actin forward 5′-GGCACCACACCTTCTACA-3′, reverse 5′-AGGAAGGCTGGAAGAGTG-3′. Incorporation of the SYBR Green dye into PCR products was monitored using an ABI Prism 7700 sequence detection system. The comparative threshold cycle (C_T_) method (ΔΔC_T_) was used for relative quantification of gene expression.

### Immunohistochemistry

Mouse tumor tissues were fixed in 10 % neutral buffered formalin for 3–6 h, maintained in 50 mmol PBS (pH 7.2) containing 5 % sucrose overnight and embedded in paraffin. Tissue sections (5 µm) were then mounted on glass slides according to the manufacturer’s instructions. The endogenous peroxidase activity was quenched by incubation of sections in 50 %/50 % solution of 3 % hydrogen peroxide and absolute methanol. Antigen retrieval was performed using 1 mM EDTA (ethylenediamine-tetra acetic acid), pH 8.0, in steamer for 30 min. The section was incubated with mouse anti-human VEGF (sc-152, Santa Cruz Biotechnology Inc, Santa Cruz, CA, USA) and anti-human bFGF (sc-79, Santa Cruz Biotechnology Inc) monoclonal antibody at 4 °C overnight and at 37 °C for 60 min; and this was followed by incubation with a biotinylated secondary antibody (BOSTER, Wuhan, China) and biotin-peroxidase complex (BOSTER). Finally, the sections were counterstained with hematoxylin.

### Western blot

Tumor tissues from A549 bearing-mice treated with IFN-α2a-NGR or IFN-α2a were obtained from antitumor studies. The tissues were homogenated in lysis buffer and then the protein concentration was measured by the bicinchoninic acid kit (CWBIO, Beijing, China). 40 µg per band of total protein were used to normalize the Western blots. The membrane was incubated with the anti-mouse-bFGF (sc-152, Santa Cruz Biotechnology Inc), anti-mouse-VEGF (1H12L17, Zymed Laboratories, San Francisco, CA, USA) antibodies for 2 h at RT and followed by incubation with specific HRP-conjugated secondary antibodies (Beijing Zhongshan Company, China) for half an hour. The immunoreactive protein was visualized with an enhanced chemiluminescence, (KGP1123, Nanjing keygen Company, China).

### TUNUL assay

DNA fragmentation induced in vascular endothelial cells by IFN-α2a-NGR or IFN-α2a treatment for 72 h was measured using the TUNEL assay (Roche Molecular Biochemicals, Mannheim, Germany) following the manufacturer's instruction. Cells were fixed with 4 % paraformaldehyde (Sigma chemicals, St Louis, USA) solution in PBS for 1 h at room temperature and rinsed with PBS, treated with 0.3 % H_2_O_2_-methanol solution, and then permeabilized with 0.25 % Triton X-100 at 4 °C for 2 min. The broken DNA ends of vascular endothelial cells were labeled with TdT and fluorescein-dUTP for 60 min at 37 °C.

### Detection of microvessel density

Paraffin-embedded sections were incubated with a monoclonal mouse anti-human CD34 antibody (Clone QBEnd/10, Neomarkers, Fremont, CA, USA) at 4 °C overnight. A positive reaction was visualized using 3, 3-diamino-benzidine as the chromagen (DAB substrate kit; Vector Laboratories Inc., Burlingame, CA, USA). Vessel density was determined by counting the number of microvessels per high-power field, as described previously (Li et al. 2011).

### Matrigel tube formation assay, migration assay and invasion assay

Matrigel (Collaborative Biomedical, Bedford, MA, USA) was added (250 µL) to each well of a 24-well plate and was left to polymerize at 37 °C for 1 h. HUVECs were seeded onto the plate at a density of 50,000 cells per well and treated with conditioned medium (containing half of the 0.2 µm filtered medium in which the lung cancer cells have been previously grown for 24 h and IFN-α2a or IFN-α2a-NGR) at 37 °C for 8 h. Tube formation was then observed using an inverted phase-contrast microscope (Nikon Corporation, Tokyo, Japan). Images were captured with a video graphic system (Optronics, Goleta, CA). Each experiment was repeated for three times. For migration assays, HUVECs were seeded onto the upper wells of a Millicell Insert (Millipore, Billerica, MA, USA) in conditioned medium containing IFN-α2a-NGR or IFN-α2a and incubated for 24 h at 37 °C. Cell migration was measured by counting the number of migrated cells in five random non-overlapped fields at 100 × magnification. Invasion assays were performed as described previously (Bonaccorsi et al. 2000). Migrated cells were quantitated by counting cells and representative images were taken using an IX70 microscope (Olympus, Tokyo, Japan) equipped with brightfield optics (200×).

### Scratch assay

The scratch assay was performed by plating cells in 6-well dishes. HUVECs were cultured in endothelial cell medium with 5 % FBS. After reaching 80 % confluence, a scratch was made through the culture dish with a sterile plastic micropipette tip to generate one homogeneous wound along each well. After wounding, the displaced cells were removed with two PBS washes. Cells were further incubated with or without IFN-α2a-NGR or IFN-α2a for 36 h and the images were taken under a microscope using an ocular grid. Three wounds were sampled for each treatment. The relative wound area was calculated as described before (Deng et al. 2006).

### Statistical analysis

Statistics were carried out with the SPSS version 11.0 (SPSS Inc., Chicago, USA). Calculations were performed using the Student’s *t* test or χ2 test where appropriate. Differences were considered significant when the *p* value was <0.05.

## Results

### Anti-tumor activity of IFN-α2a-NGR

**Fig. 1:**
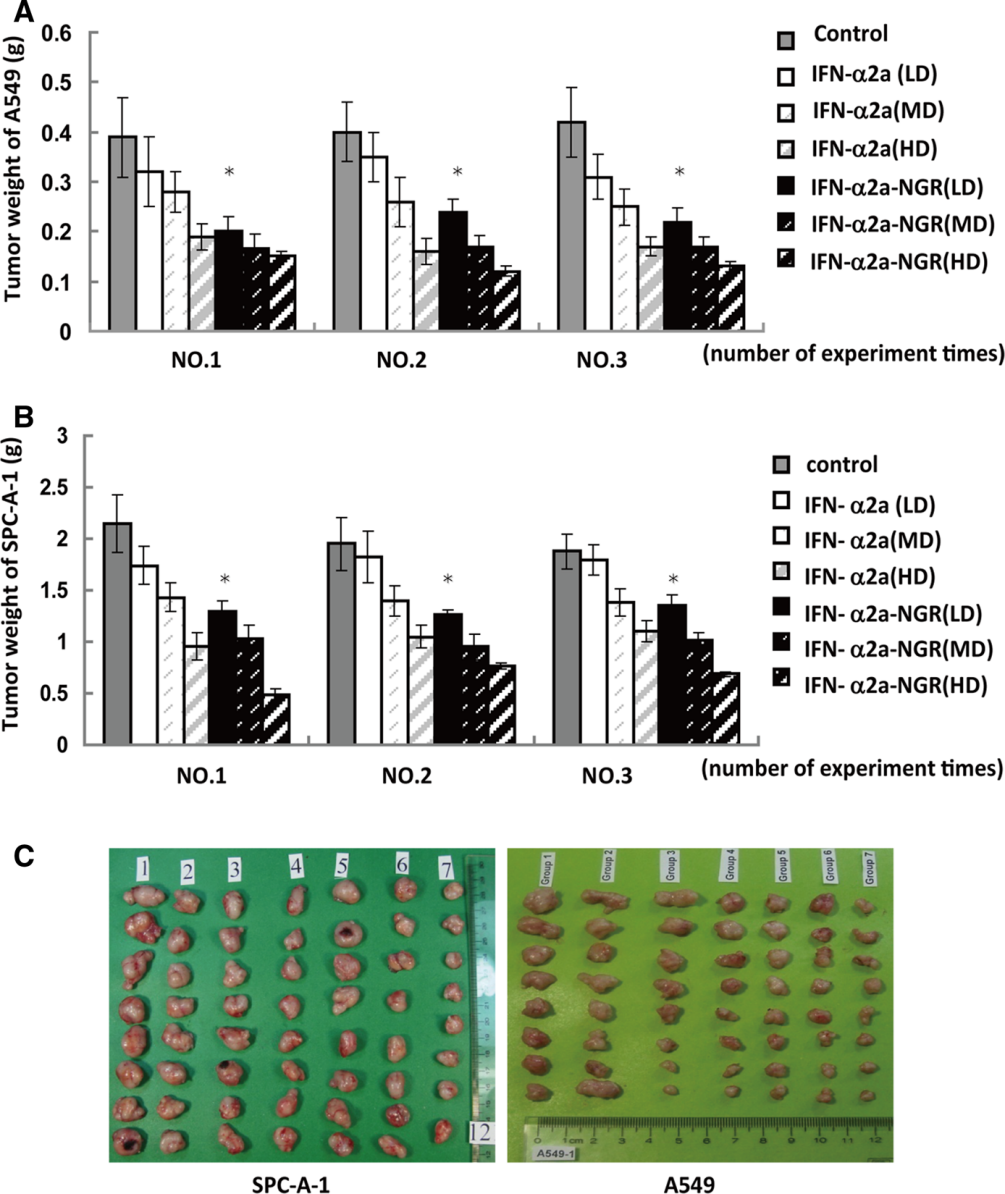
Anti-tumor effect of IFN-α2a-NGR and IFN-α2a in vivo. The anticancer activity of IFN-α2a-NGR was studied in xenografted mice. A549 cells or SPC-A-1 cells were implanted in mice, followed by daily i.p. administration with different doses of IFN-α2a-NGR or IFN-α2a, as indicated (n = 8). The mice were sacrificed and the tumor tissue was dissected. Tumor weights of different groups of A549 (**a**) or SPC-A-1-challenged mice (**b**). For all experiments, each group was performed in triplicate. Data were analyzed by Student’s *t* test. * *p* < 0.05 versus IFN-α2a groups of same doses. (**c**) Pictures of isolated tumors. NO. 1–7 represents 7 treatment groups: 1. 0.9 % sodium chloride only; 2. IFN-α2a 1 × 10^6^ IU/kg; 3. IFN-α2a 3 × 10^6^ IU/kg; 4. IFN-α2a 9 × 10^6^ IU/kg; 5. IFN-α2a-NGR 1 × 10^6^ IU/kg; 6. IFN-α2a-NGR 3 × 10^6^ IU/kg; 7. IFN-α2a-NGR 9 × 10^6^ IU/kg. Treatment with IFNα2a-NGR resulted in significant reduction in tumor volume in comparison with negative controls. In contrast, only a mild suppression of tumor growth was noted with the IFNα2a-treated mice. Similar results were obtained from both the A549 and SPC-A-1 implanted groups

To evaluate the bioactivity of IFN-α2a-NGR, we assessed its effects on tumor growth in the A549 or SPC-A-1 tumor-bearing nude mice in vivo. Pictures of isolated tumors are shown in Fig. 1c. Treatment with IFN-α2a-NGR resulted in a significant reduction in tumor weight in comparison with the IFN-α2a-treated group (Fig. 1a, b). Moreover, the anti-tumor activity of IFN-α2a-NGR at MD was similar or higher than that of IFN-α2a at HD, and the anti-tumor activity of IFN-α2a-NGR at LD was similar or higher than that of IFN-α2a at MD, suggesting that IFN-α2a-NGR was more effective in reducing tumor size in vivo.

### IFN-α2a-NGR can selectively bind to vessels of tumor tissues and decrease microvessel density (MVD)

**Fig. 2:**
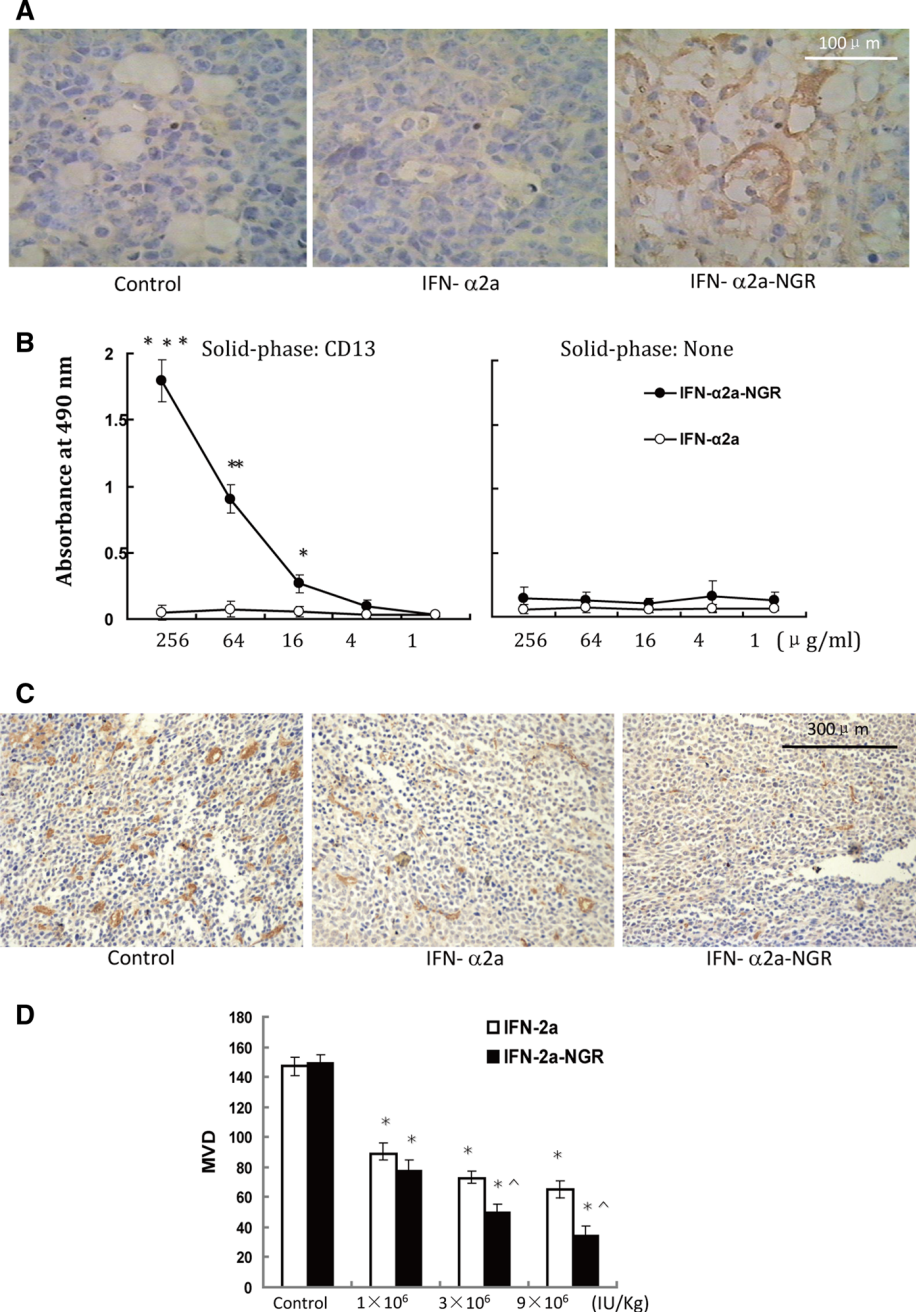
IFN-α2a-NGR can selectively bind to vessels and decrease MVD of tumor tissues. **a** Immunohistochemistry detection of IFN-α2a in tumor tissue sections. 3 × 10^6^U/kg IFN-α2a-NGR or IFN-α2a was injected to tumor-bearing mice i.v. The tumor tissue from IFN-α2a-NGR treatment group showed strong staining, whereas those from negative controls and IFN-α2a treatment groups showed weak staining with anti-human IFN-α antibody. Shown is a representative image. **b** The ELISA assay of IFN-α2a-NGR or IFN-α2a binding to microtiter plates. The plates were coated with purified human CD13. The ELISA assay was carried out with serially diluted IFN-α2a-NGR or IFN-α2a as indicated. Plates were then washed and the bound IFN-α2a-NGR or IFN-α2a was detected using an anti-hIFN-α monoclonal antibody. The bound peroxidase was detected by chromogenic reaction with o-phenylenediamine after washing with DPBS. For all experiments, each group was performed in triplicate. Data were analyzed by Student’s *t* test. Points, mean (n = 3); bars, SE. * *p* < 0.05, ** *p* < 0.01, *** p < 0.001. **c** The detection of MVD in tumor tissues by immunohistochemistry. Vascular density in the tumor sections from A549-bearing mice was determined by the most intense CD34 staining. A representative image is shown. MVD in A549 tumors treated with IFN-α2a-NGR was markedly decreased in comparison with the control and IFN-α2a-treated tumors. **d** Statistics analysis on effects of IFN-α2a-NGR and IFN-α2a treatments on MVD in tumor tissues at different doses. Single endothelial cells or clusters of endothelial cells positive for CD34 staining were considered as individual vessels. * *p* < 0.05 versus control groups, ^ *p* < 0.05 versus IFN-α2a groups

As shown in Fig. 2a, 3 × 10^6^U/kg IFN-α2a-NGR or IFN-α2a was injected to tumor-bearing mice i.v. 30 min later, mice were sacrificed and tumor tissue sections were stained with mouse anti-hIFNα monoclonal antibody. The tumor sections from the IFN-α2a-NGR treated group showed strong staining, whereas those from the negative control and IFN-α2a treated groups showed weak staining with anti-human IFN-α antibody. The tumor vessels targeting property of IFN-α2a-NGR conjugates may therefore contribute to the accumulation of IFN-α2a-NGR in the tumor tissue. As contrast, non-tumor targeted IFN-α2a showed remarkably lower concentration in tumor than IFN-α2a-NGR. Besides, the tumor tissues obtained from IFN-α2a-NGR-treated mice had a markedly higher accumulation of drugs that were localized around the tumor vessels and the surrounding areas. In addition, the binding affinity of IFN-α2a-NGR or IFN-α2a to hCD13 was assessed. As shown in Fig. 2b, the binding affinity between IFN-α2a-NGR and CD13 increased in a dose-dependent manner, while IFN-α2a displayed a lack of affinity to CD13. The binding of CD13 and IFN-α2a-NGR could be neutralized by both an anti-CD13 antibody and free NGR peptide (Figure S1). Figure 2c shows that there were new vessels of irregular lumens in tumor tissues. Every drug-treated group had a lower MVD than that of control. However, at the same dose, IFN-α2a-NGR displayed a significantly decreased MVD when compared to that of IFN-α2a (Fig. 2c, d). Besides, CD13 was highly expressed around the tumor vessels and the tumor tissue from IFN-α2a-NGR treatment group showed weak staining, whereas those from IFN-α2a treatment groups showed stronger staining with anti-CD13 antibody and control groups showed strongest staining (Figure S2).

### IFN-α2a-NGR reduced the expression of VEGF and bFGF

**Fig. 3:**
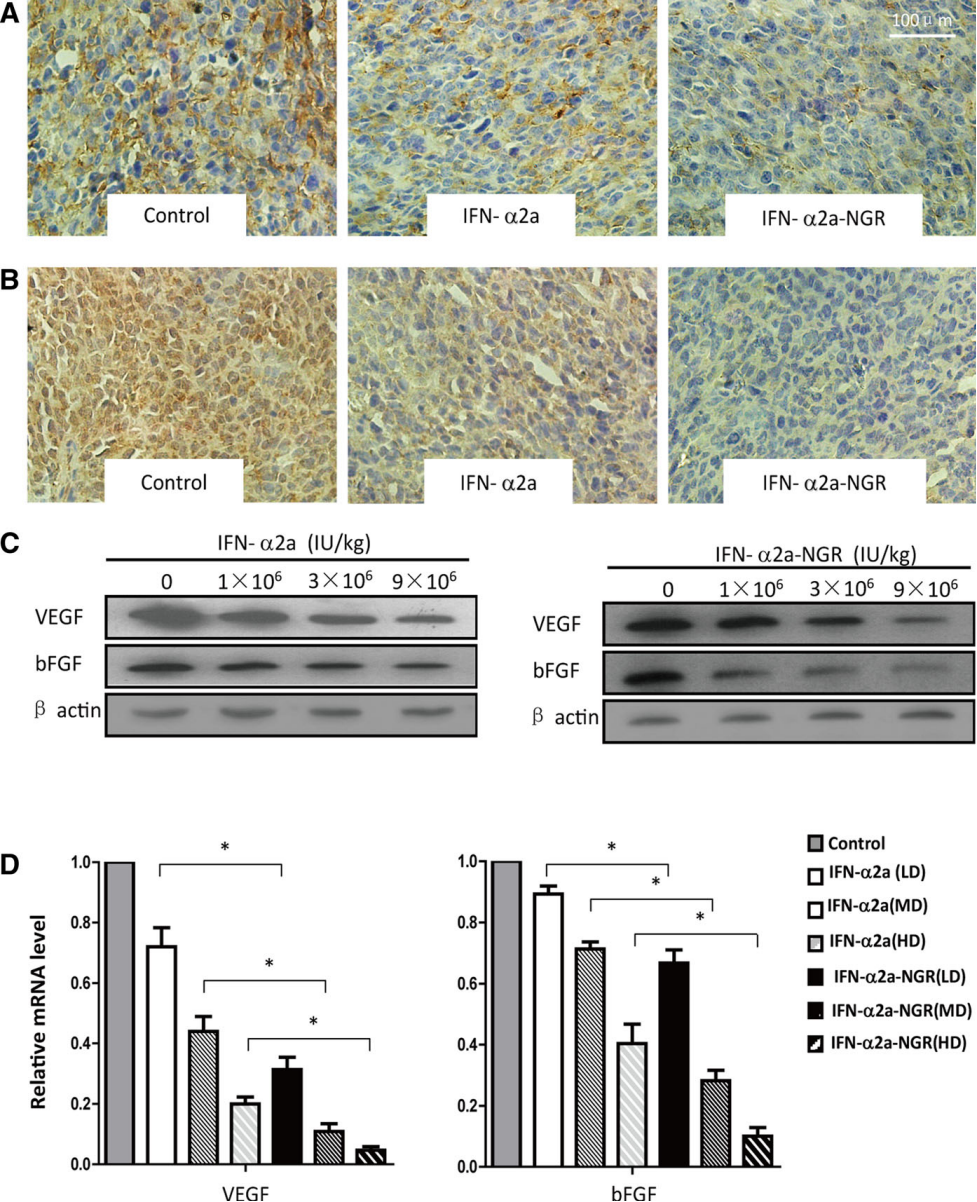
IFN-α2a-NGR decreased the expression of VEGF and bFGF. Paraffin-embedded tumor tissues from A549 bearing-mice treated with IFN-α2a-NGR or IFN-α2a were obtained from antitumor studies, prepared and coated on polylysine-coated slides. The sections were stained with the mouse anti-human- bFGF (Santa Cruz), VEGF (Zymed) antibodies to examine VEGF (**a**) and bFGF (**b**) expression in tumor tissues. (**c**) Western blot analysis of VEGF and bFGF expression in tumor tissues. (**d**) The Real-time PCR assay of VEGF and bFGF mRNA level in tumor tissues. For all experiments, each group was performed in triplicate or quadruplicate; the mean value ± SD of a representative of three independent assays is shown. Data were analyzed by one-way ANOVA statistical tests. * *p* < 0.05

VEGF and bFGF are known as tumor-derived pro-angiogenic factors which play a crucial role in tumor progression. The paraffin-embedded tumor tissues from antitumor studies were analyzed by histology staining with anti-human VEGF or bFGF. A marked reduction of VEGF and bFGF in tumors treated with IFN-α2a-NGR was observed (Fig 3a, b). As can be seen in Table 1, tissues from IFN-α2a-NGR-treated mice displayed a greater reduction in VEGF expression compared to that of IFN-α2a. Similar effects on bFGF expression were observed. Both IFN-a2a-NGR and IFN-a2a decreased the expression levels of VEGF and bFGF in tumor tissues compared to control mice (Table 1). This decrease in expression was observed at all doses except for LD (1 × 10^6^ IU/kg) IFN-α2a. Furthermore, western blot assay also proved that IFN-α2a-NGR was more potent in reducing VEGF and bFGF expression compared to IFN-α2a (Fig. 3c). A real-time PCR assay showed that there was a significant reduction in the transcription of these two factors in IFN-α2a-NGR- and IFN-α2a-treated cells compared with controls, in a dose-dependent manner. (Fig. 3d).

**Table 1:** The expression level of VEGF and bFGF in tumor tissues of tumor-bearing mice

Groups	n	VEGF			Positive ratio (%)	bFGF			Positive ratio (%)
		−	+	++		−	+	++	
Control	24	2	5	17	91.7	2	4	18	91.7
1 × 10^6^U/kg IFN-α2a	24	3	10	11	87.5	3	9	12	87.5
3 × 10^6^U/kg IFN-α2a	24	5	11	8	79.2*	7	7	10	70.8*
9 × 10^6^U/kg IFN-α2a	24	9	12	3	62.5*	9	10	5	62.5*
1 × 10^6^U/kg IFN-α2a-NGR	24	8	10	6	66.7*^	9	12	3	62.5*^
3 × 10^6^U/kg IFN-α2a-NGR	24	14	6	4	41.7*^	10	12	2	58.3*^
9 × 10^6^U/kg IFN-α2a-NGR	24	17	7	0	29.2*^	14	9	1	41.7*^

*Negative* (−) indicates that no positive cells were founded in tumor tissue sections. *Weak positive* (+) indicates a positive ratio between 0 and 50 %. *Strong positive* (++) indicates a positive ratio >50 %. Data were analyzed by χ2 tests in the SPSS statistical package. Differences were considered significant when the *p* value was <0.05

* *p* < 0.05 versus control groups, ^ *p* < 0.05 versus IFN-α2a groups

### IFN-α2a-NGR induced apoptosis and inhibited the migration, invasion and canaliculization of vascular endothelial cells

**Fig. 4:**
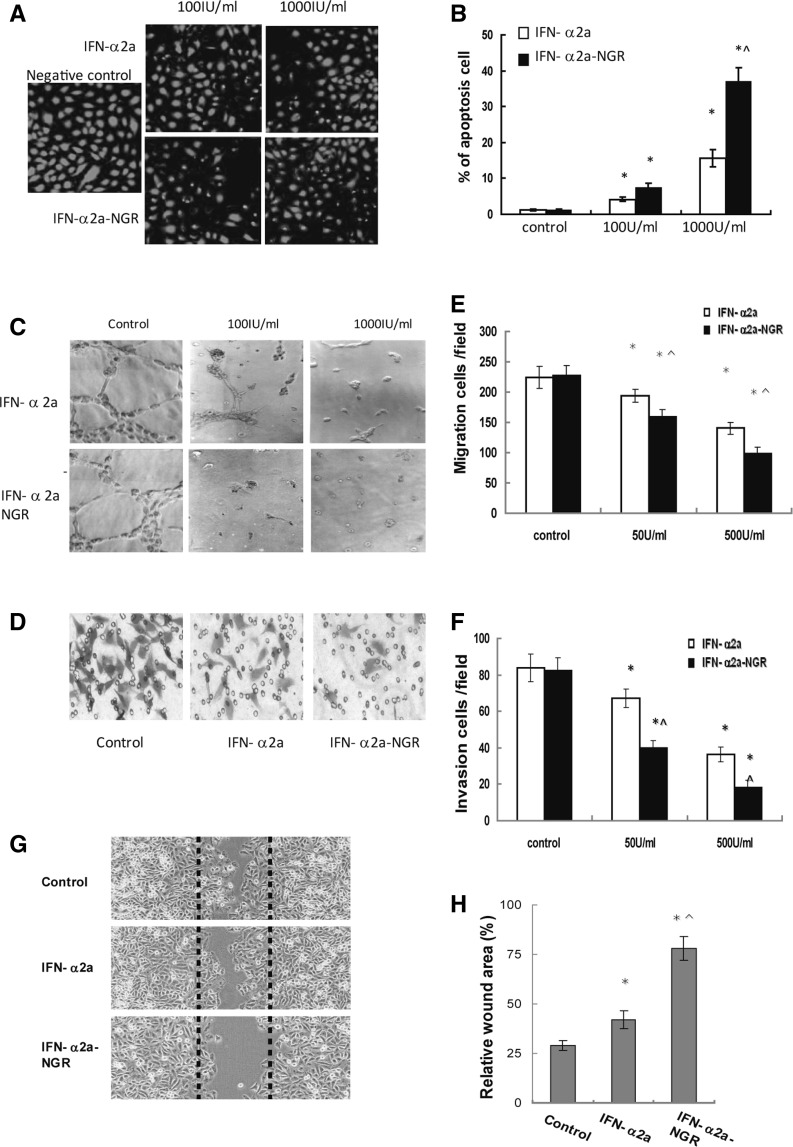
IFN-α2a-NGR induced apoptosis and inhibited the migration, invasion and angiogenesis of endothelial cells. **a** TUNEL assay of vascular endothelial cells treated with IFN-α2a-NGR or IFN-α2a (100 or 1,000 IU/mL). **b** Statistics analysis results of apoptosis ratios. Results are expressed as mean ± SD. * *p* < 0.05 versus control groups, ^ *p* < 0.05 versus IFN-α2a groups. **c** Inhibition of tube formation of EC treated with IFN-α2a-NGR was detected on Matrigel. ECs (1 × 10^5^/mL) were seeded on top of the Matrigel with complete DMEM medium containing/not containing IFN-α2a-NGR or IFN-α2a and cultured for 8 h. The representative photographs were taken using light microscopy (200 × view field). **d** EC cells were seeded in upper chamber of Millicell insert with serum-free DMEM medium containing/not containing IFN-α2a-NGR or IFN-α2a and incubated with 10 % FCS DMEM medium in the lower chamber for 12 h. The cells were fixed and stained with crystal violet. The migrated cells were visualized under a light microscope. **e** Data of **d** were analyzed by Student’s *t* test. * *p* < 0.05 versus control groups, ^ *p* < 0.05 versus IFN-α2a groups. **f** EC cells were seeded in Millicell insert coated with Matrigel Matrix. Invasion was induced by 10 % FCS DMEM medium containing 30 ng/mL basic fibroblast growth factor to the lower chamber. The cells invaded through the membrane of Millicell Inserts were counted in five random nonoverlapped 200 × view fields. The data are expressed as the mean ± SD of four separate experiments. For all experiments, each group was performed in triplicate. Data were analyzed by Student’s *t* test. * *p* < 0.05 versus control groups, ^ *p* < 0.05 versus IFN-α2a groups. **g** Light microscopic images showed the inhibited migratory ability of HUVEC cells after 36 h of treatment with IFN-α2a-NGR. The wound widths were measured under a microscope using an ocular grid at 200 × magnification. **h** The wound areas were measured 36 h post injury. The results represent mean ± SD of at least 12 wounds and were analyzed by the Student’s *t* test, * *p* < 0.05 was considered significant. * *p* < 0.05 versus control groups, ^ *p* < 0.05 versus IFN-α2a groups

To further investigate the inhibitory function of IFN-α2a-NGR on tumor neovessels, we assessed the effect of IFN-α2a-NGR on the apoptosis of vascular endothelial cells. TUNEL analysis showed significant apoptosis of vascular endothelial cells at 72 h after treatment with IFN-α2a-NGR, where apoptosis inductivity was 7 and 36 % at concentrations of 100 and 1,000 IU/mL, respectively. IFN-α2a showed a lower apoptosis induction, with 4 and 15 % at concentrations of 100 and 1000 IU/mL, respectively (Fig. 4a, b). Tube formation was significantly decreased by treatment with IFN-α2a (100 or 1000 IU/mL) or 100 IU/mL IFN-α2a-NGR (Fig. 4c). Our migration assay results showed that the mobility of the cells had reduced for 13.7 % (50 IU/mL), 29.3 % (500 IU/mL) and 37.8 % (50 IU/mL), 56.4 % (500 IU/mL) respectively, after treatment with IFN-α2a or IFN-α2a-NGR for 12 h (Fig. 4d, e). Similar findings were observed in the invasion assays, where the values had reduced by 19.1 (50 IU/mL), 52.1 (500 IU/mL), 56.2 (50 IU/mL) and 78.1 % (500 IU/mL), respectively, after treatment with IFN-α2a or IFN-α2a-NGR (Fig. 4f). To evaluate the migration ability of HUVECs in the presence of these compounds, the migration distance of cells into the ‘scratch’ region was measured after 36 h of incubation. As shown in Fig. 4g, h, migration of cells into the scratch region was markedly reduced in the presence of IFN-α2a-NGR, indicating that IFN-α2a-NGR and IFN-α2a can potently inhibit the migration and mobility of vascular endothelial cells and IFN-α2a-NGR has a stronger inhibition effect than IFN-α2a.

## Discussion

IFN therapy is used in the treatment of a number of malignant diseases. However, this therapy is associated with significant side effects. To improve the efficacy and reduce the adverse effects of IFN treatment, we combined the tumor target therapy together with IFN-α2a. NGR peptides can bind with an aminopeptidase isoform of aminopeptidase N (APN), also known as CD13, which is expressed in tumor vessels but not in normal epithelia or myeloid cells (Curnis et al. 2002a, b). CD13/APN has been shown to mediate the migration and invasion of endothelial cells in the process of angiogenesis. In previous studies, we coupled NGR with the C-terminal of IFN-α2a (Meng et al. 2007). The recombinant IFN-α2a-NGR was expressed in *E. coli.* and was purified by ion exchange chromatography. The preclinical safety evaluation of IFN-α2a-NGR showed that administration of this recombinant protein was extremely well tolerated with very high doses in mice, rats and monkeys (Meng et al. 2008). In the current study, we confirmed the tumor inhibition effect of IFN-α2a-NGR in vivo and found that it was 3× more potent than IFN-α2a in elicting its anti-tumor effects in A549- and SPC-A-1- bearing mice. This suggests that IFN-α2a-NGR could increase the therapeutic index of IFN-α2a while lowering the dose. This recombinant strategy enhanced IFN-α2a tumor therapy and may be important in reducing the concentration of drugs needed and thus reducing the potential side effects caused by high dose IFN-α2a.

Our previous study also showed IFN-α2a-NGR had the ability to bind with the tumor vessels (Meng et al. 2007). Immunohistochemistry results confirmed the high binding affinity of IFN-α2a-NGR to the tumor vasculature and further demonstrated that IFN-α2a-NGR can selectively target tumor vessels. The aggregation of IFN-α2a-NGR in tumor tissue was significantly more elevated than that in IFN-α2a-treated group after intravenous injections for 30 min, suggesting that IFN-α2a-NGR effectively improves the tumor targeting ability of IFN-α2a and raises the drug concentration in tumor tissues, and thereby increasing the anti-tumor effects in vivo.

It has been demonstrated that angiogenesis is crucial for tumor migration and invasion. The density of newly formed micro-vessels in tumors is closely correlated with an increase in the volume of tumors (Poon et al. 2002). In this study, we found that the MVD of tumor tissues in the IFN-α2a-NGR therapy group was less than that of the control and IFN-α2a-treated groups. The findings further showed that IFN-α2a-NGR has more powerful anti-tumor effect than IFN-α2a, possibly due to the tumor vessels targeting property of IFN-α2a-NGR contributing to the accumulation of IFN-α2a-NGR in tumor and enhancing the anti-angiogenesis of IFN-α2a. We speculated that NGR binding to APN might block the function of APN, which in turn contributed to the anti-angiogenesis of IFN-α2a.

VEGF is a vascular permeability factor responsible for the characteristic leakiness of tumor blood vessels and is also an important growth factor with angiogenic activity (Nagata et al. 2002). bFGF, known as an endothelial cell survival factor, is an important regulator of endothelial cell proliferation, migration, and protease production (Edelman et al. 1992). We found that IFN-α2a-NGR could down-regulate the expression of VEGF and bFGF, thus decreasing the levels of angiogenesis and contributing to an inhibition in tumor growth.

To investigate whether the decrease in MVD of tumor tissues resulting from IFN-α2a-NGR was associated with an increase in endothelial cell apoptosis, we performed TUNEL staining of HUVEC cells and found that IFN-α2a-NGR treatment significantly induced apoptosis at a higher rate than IFN-α2a. This may be due to the specific affinity of NGR to vessel endothelial cells. The tumor vessels targeting property of IFN-α2a-NGR conjugates may therefore contribute to the anti-angiogenesis effect.

Other characteristics of angiogenesis include the breakdown of the basal membrane, endothelial cell proliferation, migration and tube formation. IFN-α2a-NGR or IFN-α2a treatment displayed inhibition in endothelial cells migration, invasion and tube formation. In addition, the inhibition achieved with IFN-α2a-NGR treatment was better than that of IFN-α2a, at the same doses. These findings revealed that IFN-α2a-NGR is a more powerful anti-angiogenic agent than IFN-α2a. In conclusion, IFN-α2a-NGR has the ability to effectively target tumor vessels and then inhibit endothelial cell tube formation, migration and invasion through an induction in apoptosis and decreases in the expression of VEGF and bFGF.

The differences in effects that we observed with IFN-α2a-NGR compared to IFN-α2a may be due to the presence of the NGR motif. When Curnis et al. (2000) fused the NH_2_ terminus of TNF with the COOH terminus of peptides containing the NGR motif, the resulting NGR-hTNF conjugate could specifically bind with CD13, which is required for the development of tumor vessels. Administration of very small amounts of NGR-TNF was sufficient to enhance the antitumor activity (Curnis et al. 2002a, b). The binding of NGR-hTNF to endothelial cells elicits defined signaling pathways through both TNF receptors and CD13, which result in cell death by impairing survival and promoting apoptosis. Ellerby et al. (1999) fused CNGRC with a pro-apoptotic peptide to form a new therapeutic agent which showed selective toxic to angiogenic endothelial cells and anti-cancer activity in mice.

Our study shows that the treatment with IFN-α2a-NGR was successful and more efficacious compared to that of IFN-α2a, with its ability to target tumor vessels while preserving the original function of IFN-α2a. The tumor blood vessel ‘homing’ peptide NGR has also been demonstrated to function as targeting elements which served to improve the binding efficiency of HUVEC cells in gene delivery (Liu et al. 2000). CD13 by itself can directly lead to a highly selective homing on tumor blood vessels and apoptosis of angiogenic endothelial cells in vivo, thus significantly contributing to the control of tumor growth and accelerating the tumor angiogenesis inhibiting effect. Studies demonstrated that IFN-α treatment can induce impressive MVD reduction and tumor growth inhibition (von Marschall et al. 2003). It was reported that these anti-angiogenic activities of IFN-α associated with the regulation of endothelial cell motility and survival and with inhibition of molecules involved in the angiogenic response, such as VEGF and bFGF (Singh et al. 1995; Albini et al. 2000; Sgonc et al. 1998). NGR may contribute to the endothelial cell motility inhibition and apoptosis induced by IFN-α through facilitating the binding of IFN-α to HUVEC cells which express high levels of CD13 in response to growth factors in the culture medium (Mina-Osorio et al. 2008). Therefore, both NGR and IFN-α2a portions contribute to the biological activity of IFN-α2a-NGR vascular targeting agent in vivo. The binding of NGR to CD13 may not only lead to the homing of IFN-α2a-NGR but also stabilizes the NGR-CD13/IFN-IFNR interaction. The signaling - pathways by which IFN-α2a-NGR elicits its effects still require further elucidation. Our findings from in vitro and in vivo experiments indicated that IFN-α2a-NGR is a promising anti-angiogenic agent with more significant therapeutic efficacy and lower toxicities than IFN-α2a.

## Electronic supplementary material

Below is the link to the electronic supplementary material.

Supplementary material 1 (TIFF 6125 kb)

Supplementary material 2 (TIFF 6972 kb)
