# Supplementary figures and images for: Recombinant IFN-α2a-NGR exhibits higher inhibitory function on tumor neovessels formation compared with IFN-α2a in vivo and in vitro
Source: Cytotechnology. 2014 Jun 5;67(6):1039–50. doi: 10.1007/s10616-014-9743-y (PMC4628926; doi:10.1007/s10616-014-9743-y)

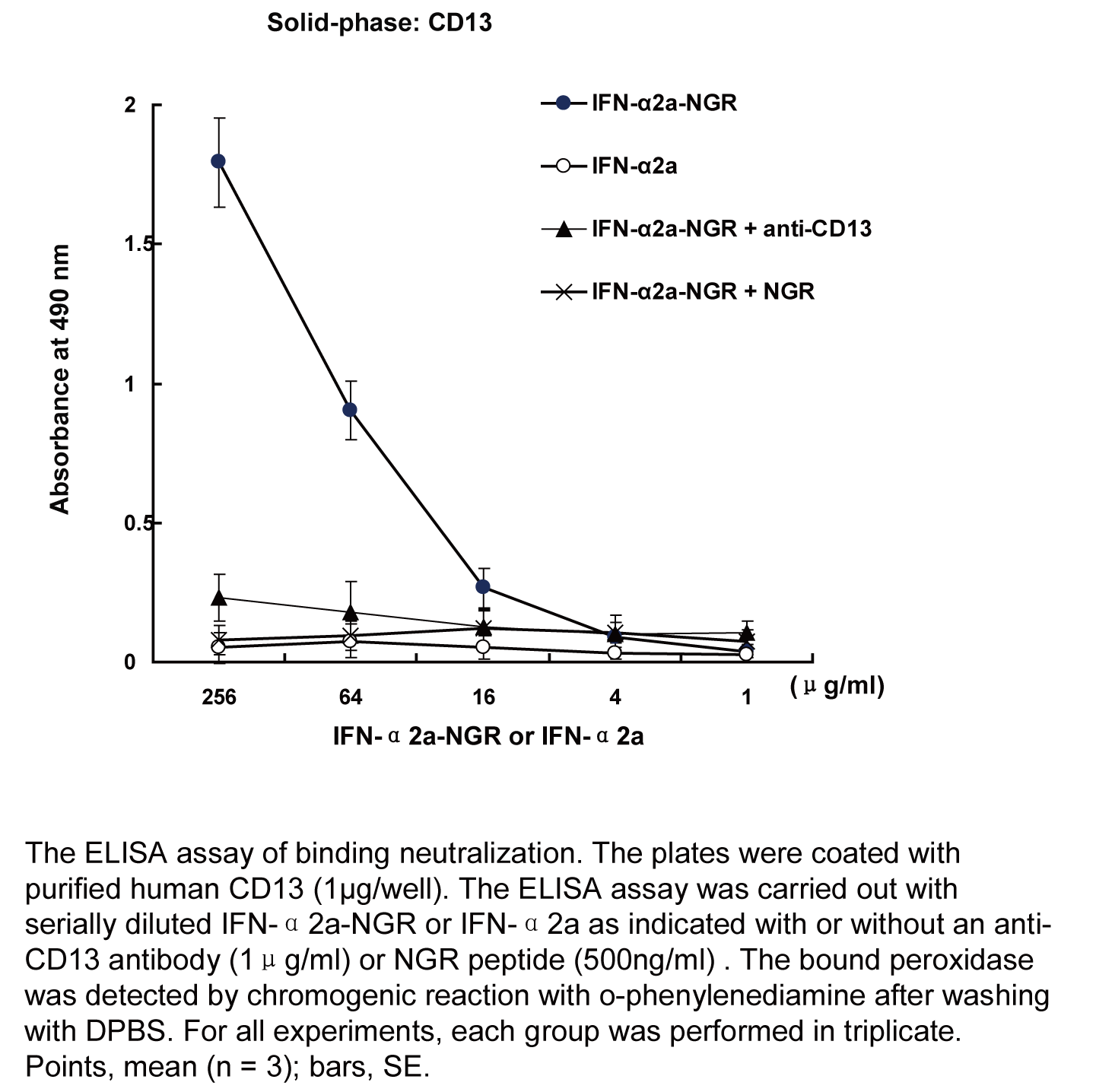

Supplement: Supplementary file 1 — Supplementary material 1 (TIFF 6125 kb) [file 10616_2014_9743_MOESM1_ESM.tif]

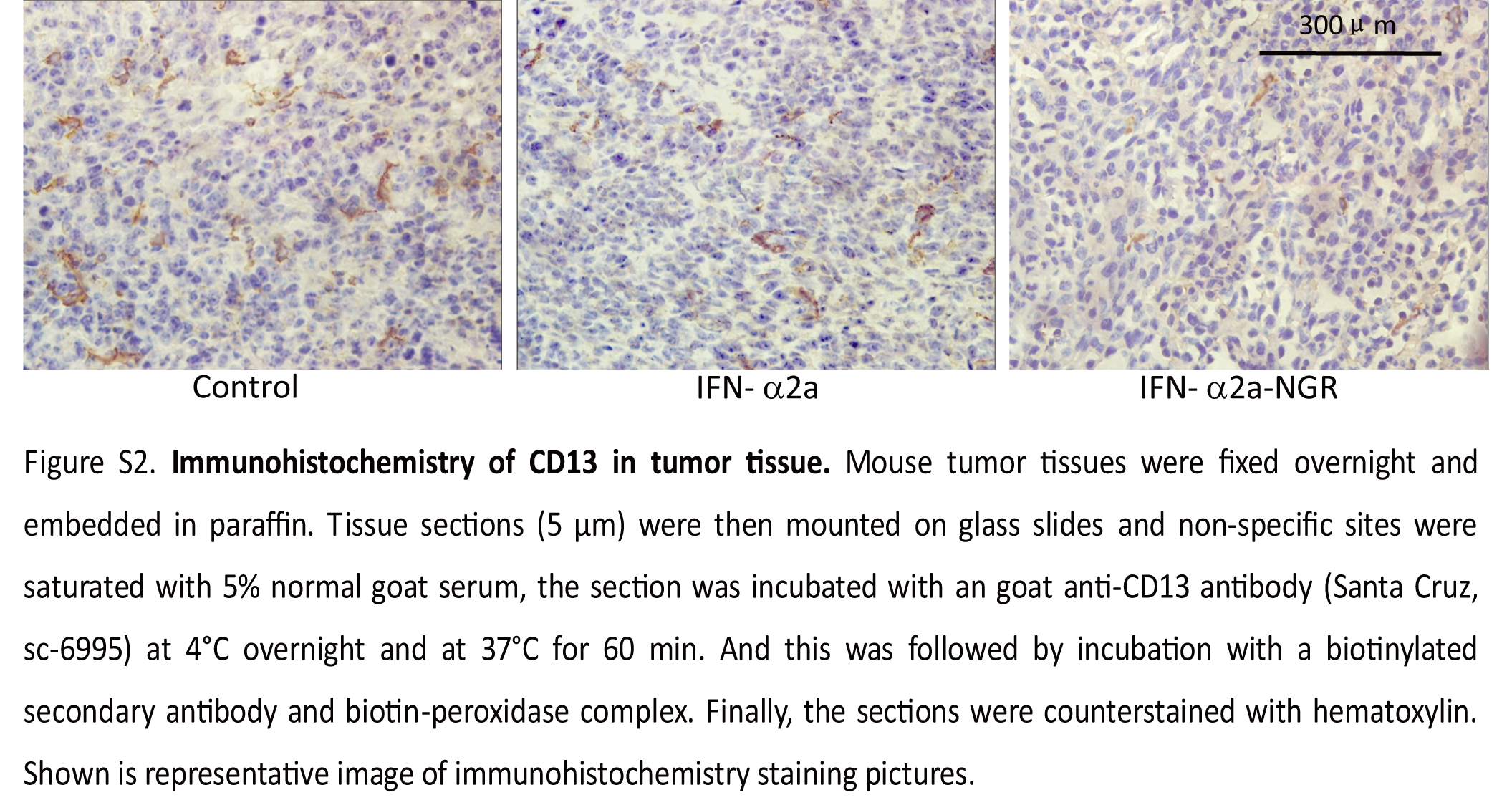

Supplement: Supplementary file 2 — Supplementary material 2 (TIFF 6972 kb) [file 10616_2014_9743_MOESM2_ESM.tif]
